# Natural Bioactive Compounds: Alternative Approach to the Treatment of Glioblastoma Multiforme

**DOI:** 10.1155/2017/9363040

**Published:** 2017-11-20

**Authors:** Vilas Desai, Alok Bhushan

**Affiliations:** Department of Pharmaceutical Sciences, Jefferson College of Pharmacy, Thomas Jefferson University, Philadelphia, PA 19107, USA

## Abstract

Glioblastoma multiforme (GBM) is the most frequent, primary malignant brain tumor prevalent in humans. GBM characteristically exhibits aggressive cell proliferation and rapid invasion of normal brain tissue resulting in poor patient prognosis. The current standard of care of surgical resection followed by radiotherapy and chemotherapy with temozolomide is not very effective. The inefficacy of the chemotherapeutic agents may be attributed to the challenges in drug delivery to the tumor. Several epidemiological studies have demonstrated the chemopreventive role of natural, dietary compounds in the development and progression of cancer. Many of these studies have reported the potential of using natural compounds in combination with chemotherapy and radiotherapy as a novel approach for the effective treatment of cancer. In this paper, we review the role of several natural compounds individually and in combination with chemotherapeutic agents in the treatment of GBM. We also assess the potential of drug delivery approaches such as the Gliadel wafers and role of nanomaterial based drug delivery systems for the effective treatment of GBM.

## 1. Introduction

Human gliomas constitute the major form of primary brain tumors [1, 2]. These tumors can be classified as low-grade gliomas, glioblastomas, or anaplastic astrocytomas, based on the degree of invasiveness and pathology of the tumor [2, 3]. The most malignant form of astrocytoma is called glioblastoma multiforme (GBM), typically characterized by an increased angiogenesis, invasion of normal brain tissues, and necrosis, and has the worst prognosis [4–6]. At a cellular level, GBM is poorly differentiated, with round or pleomorphic cells that are multinucleated and anaplastic [6–8]. Depending on origin, it is categorized as either primary GBM, which arises de novo from the glial cells, or secondary GBM, which arises from preexisting lower grade astrocytoma [6, 9]. The hallmark feature of primary GBM is overexpression of the EGFR gene and loss of heterozygosity of PTEN while secondary GBM exhibits loss of p53 and overexpression of PGDF [8–10]. Although the causes of GBM are relatively unclear, its risk may be increased by certain factors (i.e., family history, brain trauma, and immune system alteration) and conditions (i.e., Li-Fraumeni's syndrome) [5, 6]. The current therapy includes surgical resection of the tumor, radiotherapy, and adjuvant chemotherapy with temozolomide [3, 9, 11]. The complete surgical resection of gliomas is difficult, and due to local invasion and infiltration of normal tissue the tumor recurs leading to death of patients with glioblastoma [3, 9]. The efficacy of chemotherapy is further decreased by the presence of the blood brain barrier that limits the delivery of chemotherapeutics to the brain [9, 11]. The current therapy minimally improves the median survival time of patients from 12 months to ~14.6 months. The 5-year survival rate of treated patients is <10% [1, 3, 9]. Hence, there is an imperative need for the development of novel, targeted, and effective therapies for GBM.

## 2. Natural Compounds in Cancer

Conventional cancer therapies such as chemotherapy and radiotherapy essentially exert their cytotoxic effects by damaging the DNA of cancer cells [12, 13]. However, limitations exist with these treatments when used as single modalities due to the high heterogeneity in solid tumors and the deregulation of several cell signaling cascades [14–18]. GBM is particularly difficult to treat because of the heterogeneity of the tumor, its highly aggressive infiltration into the surrounding tissues, and the presence of blood brain barrier [3, 4]. Multimodality treatment approaches can be an effective strategy for GBM wherein different therapies or therapeutic agents with distinct molecular mechanisms are combined to exert an improved cytotoxic effect on cancer cells [10, 11]. In particular, multimodal therapy can work effectively by sensitizing cancer cell DNA by one agent to the damaging effects of the other [12, 13, 19].

In recent years, several epidemiological studies have investigated the role of natural, dietary compounds in influencing the development, progression, and metastasis of cancer [19–22]. A wide range of natural compounds have been recognized for their antioxidant nature and for their cancer chemopreventive potential, including soy isoflavones, curcumin, epigallocatechin, resveratrol, and retinoids [23–25]. The main objective of this review paper is to discuss the role of these natural compounds in enhancing the efficacy of chemotherapeutic drugs in GBM treatment as reported by a number of studies. We also review the significance of novel drug delivery methods such as the Gliadel wafers and the emerging role of nanomedicine in drug delivery for GBM treatment.

### 2.1. Isoflavones in Glioma

Several studies have demonstrated the benefits of consumption of a plant-based diet of fruits and vegetables [13–19]. Soy isoflavones, such as genistein, daidzein, and biochanin A, are natural polyphenolic compounds with potent antioxidant, anti-inflammatory, and weak estrogenic properties [18, 19]. Isoflavones are usually derived from soy and soy-based products but are also found in chick peas, nuts, grain products, and red clover [12, 15]. They have been implicated in cancer prevention, based on the epidemiological reports that South Asian populations have heavy consumption of soy and soy-containing foods and low incidence of cancer and cardiovascular diseases compared to the people in Western civilizations [14, 19]. Furthermore, they also reportedly have beneficial effects in endocrine-responsive cancer, osteoporosis, menopause, and coronary heart diseases [21, 22]. Interest in soy isoflavones has been renewed by the research showing the estrogen-like ring in their structure, indicating that they may be a better alternative to synthetic selective estrogen receptor modulators (SERMs) currently used in breast cancer prevention and hormone replacement therapy [20, 21, 23].

The intestinal microflora in the body converts isoflavones to isoflavonoids which reportedly play a critical, preventive role in mutation and promotional phases of cancer progression [21, 22]. Cancer chemopreventive properties of isoflavones in improving the efficacy of chemotherapy and radiotherapy have been demonstrated* in vitro* and* in vivo*, and a number of clinical trials are focusing on the anti-invasive and antiangiogenic properties of these compounds for treatment of GBM [18, 21, 23].

#### 2.1.1. Genistein

Genistein is one type of soy isoflavone that has shown promise as a chemopreventive agent due to its antioxidant and anti-inflammatory properties and its ability to potently inhibit angiogenesis and metastasis [16, 18, 20]. Our previous studies have reported its ability to inhibit invasion in a coculture model of GBM by inhibiting tyrosine kinase EGFR [24]. Other studies have shown that genistein prevents the hypermethylation of promoter regions of tumor suppressor genes (i.e., p21, BRCA1) by inhibiting DNA methyltransferases in different cancer types [20, 22]. In other research, genistein inhibited the expression of apurinic/apyrimidinic (AP) endonuclease 1 (APE1) in prostate cancer cells in a dose dependent manner [18, 23]. APE1 is an enzyme involved in the DNA base excision repair (BER) and redox signaling, and elevated levels have been correlated to resistance to chemotherapy [13, 15, 16]. Genistein is also a known inhibitor of protein tyrosine kinase (PTK), and it competes with ATP to bind to the tyrosine kinase domain thereby inhibiting the activation of tyrosine kinase-mediated downstream signaling processes [20, 22, 24]. A recent study on genistein showed its inhibitory effects on telomerase activity and subsequent cell cycle arrest in radiosensitive brain tumor cells [15, 17, 22]. There have been several clinical studies at phases I, II, and III on genistein as an adjuvant compound on patients undergoing chemotherapy or radiotherapy for prostate, bladder, or breast cancer [25]. In a study on 20 prostate cancer patients treated with pure genistein, it was observed that there were no genotoxic effects or any change in micronuclei number and no damage to the chromosomes compared to the nontreated lot [26]. A wide range of clinical studies on isoflavones have demonstrated a good safety profile even at maximal dose which is encouraging for the population [26]. However, in clinical studies, various factors such as the stage or type of cancer and differences in the metabolic state of individuals play a critical role in determining the outcome of such studies. Nonetheless, the results of a number of studies on isoflavones in cancer are encouraging and hold a potential for further research.

#### 2.1.2. Biochanin A

Biochanin A, a bioactive isoflavone and a prodrug or methoxy form of genistein that is found in red clover, has been shown to inhibit the incidence and growth of LNCaP xenograft tumors in athymic mice [21, 23]. Earlier studies showed that it has chemopreventive and anticancer potential against glioblastoma, breast cancer, prostate cancer, and oral cancer cells [11, 17, 23, 24]. Biochanin A is documented to be less mutagenic than genistein, and hence it may be a more suitable candidate for use as a chemopreventive agent [14, 18]. It is rapidly degraded to genistein, genistein conjugates, and biochanin A conjugates, which confer anticancer properties to the compound [20, 23]. It has tyrosine kinase inhibitory properties that are weaker than those of genistein and inhibits the growth of breast, colon, and prostate cancer cells* in vitro* [11, 17, 18, 27]. Studies have also shown that biochanin A has inhibitory potential on the development of lung tumors induced in mice by benzo(a)pyrene [23].

Both biochanin A and genistein have been found to inhibit both serum and EGF-stimulated growth of human prostate cancer cells [17, 18]. Our earlier studies showed that genistein and biochanin A inhibit the invasion of glioblastoma cells by inhibiting matrix metalloproteases (MMPs) [11]. Both isoflavones have also been found to enhance the efficacy of rapamycin in inhibiting the mTOR pathway in glioblastoma cells and their rapamycin-induced feedback upregulation of AKT [24].

### 2.2. Resveratrol

Resveratrol (*trans*-3,4′,5-trihydroxystilbene) (RSV) is a naturally occurring polyphenolic phytoalexin present in grapes, mulberries, peanuts, and vegetables [27, 28]. It has attracted substantial attention in recent years due to its antioxidant and anti-inflammatory properties, which give it efficacy in treating cardiovascular diseases, and due to its neuroprotective effects in penetrating the blood brain barrier, which give it efficacy for treating ischemia and hypoxia [29]. The chemopreventive and antioncogenic effects of RSV have been reported in several cancer types [27–29]. Researchers found that RSV prevented the development of skin cancer in mice during the different stages of carcinogenesis [27] and inhibited DMBA-induced mammary carcinogenesis [28]. Clinical trial studies in patients with colorectal cancer have shown that RSV hinders tumor cell proliferation and increases caspase-3 in the malignant hepatic tissue compared to the placebo patients [30, 31]. In a phase I trial study in healthy volunteers, it was observed that ingesting RSV (0.5 to 5 g/day for 29 days) led to a decrease in systemic IGF-1 and IGFBP-3 levels, which may contribute to the antiproliferative activity of RSV [32].

In earlier studies by Gangemi et al., RSV was found to induce apoptosis by activating caspase-3 in a human glioma cell line U251 [33]. Further, Ryu et al. demonstrated that RSV reduced the invasion in U373MG glioma cells induced via TNF-*α*, by regulating the NF-*κ*B activation and expression of upa/upar [34]. A study by Jang et al. reported the chemopreventive and anticarcinogenic effects of RSV in the different stages of carcinogenesis, namely, initiation, promotion, and progression [28]. RSV causes cell death in GBM cells through mechanisms such as autophagy, apoptosis, and senescence [28, 35]. It exerts its cytotoxic and cytostatic effects in cancer cells by modulating the cell cycle at specific points including an S phase arrest in medulloblastoma cells [35].

A study by Filippi-Chiela et al. on several GBM cell lines found that RSV potentiates the toxicity of TMZ in combination treatment mainly by inhibiting TMZ-induced G2/M arrest followed by induction of senescence and MC [35]. This G2/M arrest was p53 independent, as it was observed in all glioma cells tested, including p53 mutant cells U251 and U138 [35]. Cilibrasi et al. investigated the effects of RSV on seven glioma stem cell (GSC) lines derived from GBM patients. They observed that RSV inhibited the cell proliferation, increased cell mortality, and reduced motility of the cells by modulating the Wnt signaling pathway [36–38]. These findings clearly suggest that RSV possesses potent therapeutic properties and may work as effective adjuvant molecules in combination therapy of cancer.

### 2.3. Epigallocatechin Gallate

Epigallocatechin gallate (EGCG) is a major polyphenolic green tea component and a major catechin in green tea [39, 40]. EGCG has been extensively investigated for its potential chemopreventive and chemosensitizing properties in a variety of malignant cancers [41, 42]. It has the ability to bind to GRP78, a key prosurvival component of ER stress response system, and inactivate its antiapoptotic function, an interaction that makes cancer cells more chemosensitive [41]. A number of studies have shown that EGCG increases the sensitivity of different cancer types to different apoptotic drugs, such as 5-fluorouracil, gemcitabine, or taxol,* in vitro* [42–44] and to doxorubicin and paclitaxel* in vivo* [44, 45]. Studies have also reported that EGCG can reverse drug resistance by inducing apoptosis and inhibiting P-gp expression and ABCG2 in drug-resistant cancer cells of the ovaries, breast, and lung [40, 42]. There have been reports of the inhibition of Wnt signaling by EGCG in breast cancer cells and an upregulation of p53 transcriptional activity in LNCaP cells [42, 46, 47]. In a double-blind placebo-controlled clinical trials with green tea catechins (GTCs) against prostate cancer, it was shown that GTCs were safe and effective in treating premalignant lesions before the prostate cancer developed [48]. In another phase II study among 42 patients of androgen independent prostate carcinoma, a minimal antineoplastic activity with a decline in prostate specific antigen (PSA) levels was reported [49]. However, there have been a few positive reports with EGCG inducing apoptosis in leukemic B-cells in majority of patients of chronic lymphocytic leukemia (CLL) and patients with low-grade B-cell malignancies [50].

A recent study by Zhang et al. reported that EGCG reduced U87 and C6 GSLC (Glioma Stem-Like Cells) viability, neurosphere formation capability, and migration. EGCG also sensitized GSLCs to temozolomide, a phenomenon associated with downregulation of P-gp* in vitro* [40, 43]. EGCG was observed to induce apoptosis in U87 GSLCs by reducing Akt phosphorylation, inactivating antiapoptotic protein Bcl-2, upregulating the apoptosis-promoting protein Bax, and cleaving PARP [51, 52]. Since gliomas are sensitive to apoptosis through the downregulation of Bcl-2 family [47, 51], EGCG is potentially an effective molecule to target GSLCs associated with inhibition of an Akt-related pathway [52]. Although it remains unclear whether EGCG can achieve chemosensitization of cancer cells across the blood brain barrier, which would be required for the treatment of malignant gliomas, these studies provided clear evidence that polyphenol compounds like EGCG can significantly augment the chemotherapeutic efficacy of cancer drugs in a combination treatment both* in vitro* and* in vivo*.

### 2.4. Retinoids

Retinoids have also been found to potentially enhance the efficacy of chemotherapy and radiotherapy in GBM. Retinoids are a class of chemical compounds that are related to vitamin A and are fat soluble [53, 54]. Retinol is transported from the liver to the target tissues as retinol-binding protein and is then enzymatically converted to retinaldehyde and consequently to retinoic acid [53, 55]. The retinoid signaling pathways play an important role in neurogenesis, dendritic growth of hippocampal neurons, and higher cognitive functions [53, 54]. Studies have shown that retinoids strongly inhibit the cell proliferation and migration in primary cultures of human glioblastoma multiforme [54, 55]. Retinoids can successfully induce differentiation but cannot induce apoptosis. However a synthetic analog of all-*trans* retinoic acid (ATRA), N-(4-hydroxyphenyl) retinamide (4-HPR), exhibits both antiproliferative and proapoptotic effects [55]. 4-HPR is a much more effective and relatively less toxic compound than ATRA, and it is proven to be effective even in ATRA-resistant cells [55, 56].

In one notable study in which 4-HPR treatment was used in combination with the knockdown of survivin, a prosurvival protein overexpressed in GBM that has been positively correlated with GBM cell proliferation, around 80% of the cells were apoptotic and* in vivo* angiogenesis studies showed a noticeable decrease in tumor vascularization [54, 56, 57]. In a similar study, when GBM cells were treated with retinoids, differentiation of astrocytes was induced and telomerase activity was inhibited, allowing for an increased sensitivity to interferon-*γ* therapy [58]. Moreover, treatments with retinoids have been shown to reduce levels of inflammatory factors, potentially making the GBM cells sensitive to radiotherapy [53, 54].

### 2.5. Other Notable Natural Compounds with Promising Anticancer Activity against GBM

#### 2.5.1. Cannabis and Cannabinoids

*Cannabis sativa* L. and its derivative compounds cannabinoids have been reported to have a broad range of pharmacological effects mediated specifically by two plasma membrane receptors (CB1 and CB2) [59–62]. Δ^9^-Tetrahydrocannabinol (THC) is the most potent and abundant endocannabinoid in cannabis that can bind to and activate specific cell receptors [60, 63]. Several studies have reported the efficacy of cannabinoids in the treatment of pain, inflammation, depression, neurological disorders, and cancer [59, 60, 62–66]. The anticancer potential of cannabinoids against gliomas may be mediated through the CB1 receptor which is densely expressed in the brain as a seven-transmembrane domain G protein-coupled receptor and is activated by the receptor agonist Δ^9^-THC* in vitro* and* in vivo* in the treatment of glioblastoma (GBM) [61–66]. The activation of CB1 and CB2 by Δ^9^-THC impairs cancer cell proliferation and invasion, induces apoptosis by ceramide accumulation in culture, and also reduces the tumor volume in animals [59, 63]. Δ^9^-THC in combination with temozolomide (TMZ) has shown a robust anticancer activity in TMZ-sensitive as well as TMZ-resistant tumors in glioma xenografts [65]. A phase I clinical study in brain tumor patients has reported the safety of direct intratumoral injection of tetrahydrocannabinol in recurring GBM [66]. However, to validate the efficacy of cannabis in preventing gliomas and the methods of administering the other derivatives of cannabis safely, more clinical trials are needed [66]. In recognition of the promising anticancer potential of Δ^9^-THC in the preclinical studies with a fair safety profile, it has become an important therapeutic target for the treatment of GBM and has also prompted a human clinical trial [61, 66].

#### 2.5.2. Neurostatin

Neurostatin is a natural glycosphingolipid, an O-acetylated ganglioside GD1b, present in the mammalian brain, and shows strong inhibition of astroblast and astrocytoma division [66, 67]. Although it exhibits a high inhibitory activity against gliomas, it is relatively less abundant in the brain [68, 69]. However, neurostatin has now been extensively purified from the ganglioside extracts of rats and bovine and porcine brain and is observed to be cytostatic against C6 glioma cells and grade III and IV human astrocytoma cells [66, 69]. In particular neurostatin has been shown to impair glioma cell proliferation* in vivo *by inducing cell cycle arrest and potentiating the immune cell response to the tumor through the activation of CD4+ and CD8+ lymphocytes [67]. Valle-Argos et al. reported that the anticancer activity of neurostatin* in vitro* and* in vivo* in gliomas is mediated through the arrest of cell cycle progression, while interfering with the angiogenic and invasive mechanisms [66, 67]. Neurostatin was shown to inhibit the expression of cell cycle promoters (cyclins) and CDKs while upregulating cell cycle inhibitors such as p21 and p27 [67, 70]. The promitogenic pathways of MAPK and PI3K were also reportedly blocked through the inhibition of EGFR signaling [67, 70]. Gangliosides like neurostatin are ubiquitous molecules with potent and specific biological actions, and the preclinical studies strongly indicate that it is a promising therapeutic candidate in the treatment of gliomas [69, 70].

#### 2.5.3. *Bipolaris setariae* Fungi

*Bipolaris* is a genus of dematiaceous hyphomycetes with more than 100 species [71]. Ophiobolin A (OP-A), a sesterterpenoid that is produced by the plant pathogenic fungi, was purified from the culture extract of* Drechslera gigantea* and characterized to be an effective phytotoxin [72, 73]. Further studies on the compound showed a broad spectrum of biological and pharmacological characteristics including anticancer activity [72, 73]. A number of studies have reported the anticancer activity of OP-A with an IC_50_ in micromolar concentration range against different glioblastoma and neuroblastoma cells [73, 74]. A study by Bury et al. reported that OP-A caused marked changes in the organization of the actin cytoskeleton and induced paraptosis through the disruption of internal potassium ion homeostasis in glioblastoma cells [73]. The reported studies indicate that the OP-A causes mitochondrial dysfunction and ER stress, impairs cell cycle progression, and inhibits multiple oncogenic signaling pathways in glioblastoma cells [73, 74].  Table 1 shows some of the natural compounds used in the treatment of GBM and their reported mechanism of action in* in vitro* and* in vivo* studies. OP-A thus represents a class of natural compounds that can be used to combat cancer types exhibiting different levels of resistance to proapoptotic stimuli [72, 74].

## 3. New Approaches to Drug Delivery

### 3.1. Gliadel Wafers

Despite surgical resection of GBM tumor followed by radiotherapy and chemotherapy (i.e., carmustine (BCNU) and temozolomide), recurrences are inevitable [75, 76]. Effective delivery of drugs to the tumor is a major challenge due to systemic toxicities and the need for transport across the blood brain barrier [75–77]. Gliadel wafers offer a novel system for delivering chemotherapeutic agents to GBM cells. Approved for treating recurrent glioblastoma by FDA in 1995, Gliadel wafers are biodegradable polymers loaded with carmustine [77, 78]. They are implanted at the resection site, and the drug is gradually released over a period of 2-3 weeks as the wafer polymer degrades [78–80]. In a placebo-controlled, double-blind phase 3 trial in 2003, 2-year survival was higher among glioblastoma patients treated with Gliadel wafers (15.8%) than patients who received placebo (8.3%); median survival was 13.8 months in the patients treated with the Gliadel wafer and 11.6 months in the placebo group [79–81].

Another study treated glioma patients who had undergone surgery with Gliadel wafers soaked with temozolomide [80, 81]. The patients treated with Gliadel wafer and temozolomide survived 20.7 months (median) and the 2-year survival rate was 36% [80]. Several other studies have reported that when Gliadel wafers are implanted in the resection cavity of malignant gliomas, local drug delivery is improved and systemic side effects are reduced in recurrent glioma treatment. However, there have also been reports that use of Gliadel wafers resulted in adverse effects, including cerebral edema, surgical site infection, perioperative seizures, and severe hydrocephalus leading to death [77–79, 82]. Hence, the practice of using Gliadel wafers for the treatment of GBM needs to be reevaluated.

### 3.2. Nanomedicine and Drug Delivery across the Blood Brain Barrier

During the last couple of decades, nanomedicine has progressed significantly, especially in the field of cancer therapeutics. A key feature of the nanomaterials is their size which operate at the same scale as the biological molecules and pathways. This means that nanoscale materials can be designed to interact with biological entities in a direct, efficient, and precise manner and that they can help us understand biological processes and pathways at the molecular level [83, 84]. Nanometerials can be designed to circumvent or cross the BBB and hence may serve as an effective drug delivery system in GBM treatment.

Treatment for GBM is often impaired because therapeutic agents cannot be delivered adequately to the target tumor cells; the problem is further compromised by the presence of blood brain barrier (BBB) [85, 86]. Blood brain barrier (BBB) is a unique anatomical structure mainly formed by tight junctions and adherence junctions between the brain endothelial cells that selectively regulate the flow of ions, nutrients, and cells into the brain [85, 87]. This restricted permeation of drugs to the GBM cells, allowing a fraction of cells called cancer stem cells (CSCs) to evade drug cytotoxicity and develop therapeutic resistance [86, 88, 89]. Further, the poor delivery of chemotherapeutic agents can also be attributed to their large size, hydrophobic nature, and their efflux by the MDR efflux pumps expressed by BBB and tumor cells [87, 88]. Thus, targeted, effective, therapeutic regimens are needed that can cross the BBB and reach their target in the brain [90, 91]. A few proposed strategies include passive permeation of lipidated drugs, development of prodrugs that can hijack the transport mechanism of BBB, and drug-loaded nanocarriers [87, 88, 92, 93].

Apart from a limited number of liposoluble, small molecules, most of the other molecules need a specific transport system to cross the BBB [84, 92]. Hence, a more selective and targeted approach such as the nanoparticles based system can be employed to design targeted therapies. Colloidal systems such as the nanoparticle system allow for the design of nanocarriers with surface properties to overcome the biochemical/biophysical barriers, and can be tailored to deliver the drugs across the BBB. Moreover, the surface properties of the nanocarriers can be modified for a selective, controlled drug release with minimal side effects and increased efficacy [84, 94, 95]. Nanomaterial based chemotherapeutic agents such as the liposomes, dendrimers, or polymeric micelles are reported to circumvent the BBB and reach the target site of action [87, 88, 96, 97]. Several nanomaterials-based drugs have been evaluated for the treatment of GBM and other cancers.  Table 2 features some of these drugs which have been approved by the regulatory authority and are in clinical use for GBM as well as other types of cancers [85–100].

Glioblastomas exhibit a high degree of heterogeneity and the integrity of BBB varies, with high-grade gliomas showing a leaky BBB and low-grade gliomas showing an intact BBB [98–100]. Hence, the GBM therapies need to be designed to identify disease-related changes in the BBB and to tailor the drug or drug nanocarriers accordingly [99, 100]. Ideally, the carrier agents should be cationic for optimal vascular absorption, small, stable in biological fluids, and tailored to carry a large drug payload [97, 99, 100]. The physiological and pathophysiological status of the gliomas also needs to be considered for optimization of any chemotherapeutic agent for GBM.

## 4. Conclusions and Future Directions

The current standard therapy of GBM has shown little promise and there have been efforts to design novel, targeted, effective therapeutics. Moreover, the current chemotherapy may also be a cause of drug resistance in GBM treatment as it severely destabilizes the cell metabolism and cell signaling network. Here we have reviewed a number of studies that report the role of various natural, dietary compounds that show significant improvement in the efficacy of chemotherapeutics in combination therapy of GBM both* in vitro* and* in vivo*. Glioblastoma is a highly heterogeneous and often fatal cancer, and attacking its chemoresistance is a critical step in combating its growth. Several of the studies reviewed emphasize the relative nontoxicity of the natural compounds and improvement in efficacy of combination therapy at a lower dosage level. We have also reviewed the role of Gliadel wafers after surgery in the local delivery of drugs such as BCNU and temozolomide in brain tumor treatments. The main challenge of GBM treatment is the inadequacy of drug delivery due to the presence of BBB. We have discussed the role of nanocarriers and nanomedicine in overcoming the obstacle of BBB to improve the efficacy of drug delivery and thus the GBM treatment. The studies reviewed above clearly demonstrate the anticancer potential of these natural compounds and indicate that they can be an alternative approach to a more effective and relatively nontoxic GBM treatment.

## Figures and Tables

**Table 1:** Natural compounds and their mechanism of action in GBM and other cancer cells.

Natural compounds	Target mechanisms
Isoflavones (biochanin A, genistein, etc.)	Inhibits MMPs and thus invasion & metastasis. Inhibits tyrosine kinase mediated downstream signaling.

Resveratrol	Induces apoptosis by activating caspase-3. Modulates cell cycle at specific points.

Epigallocatechin A (EGCG)	Binds to GRP78 (prosurvival component of ER stress response system) and inactivates its antiapoptotic function. Inhibits P-gp expression thus reversing drug resistance in cancer cells.

Retinoids	Reduces levels of inflammatory factors making cancer cells sensitive to radiotherapy. Increases sensitivity to specific therapies such as interferon-*γ* therapy by inhibiting telomerase activity.

Cannabis	Induces apoptosis by sustained accumulation of ceramide that also upregulates the ERK activity. Causes ER stress and inhibition of pAkt/mTOR leading to autophagy-mediated cell death.

Neurostatin	Inhibits cell cycle progression (suppresses cyclins and CDKs and promotes inhibitors such as p21 & p27). Impairs promitogenic pathways of MAPK and PI3K by blocking EGFR signaling.

*Bipolaris setariae* fungi	Disrupts the potassium ion homeostasis causing mitochondrial dysfunction and ER stress. Impairs cell cycle progression.

**Table 2:** Nanomaterial-based drugs in clinical use for the treatment of GBM.

Name	Nanocarrier	Characteristics	Permeability to BBB
Doxil	PEGylated liposome/doxorubicin hydrochloride	Inhibits nucleic acid synthesis, ROS	No
Abraxane	Nanoparticle albumin-bound paclitaxel	Microtubule stabilizing	No
Carbon nanomaterials (carbon nanotubes (CNTs), graphene oxide)	CNTs, nanographene oxide	DNA damage by ROS, peroxidation	No
Gold nanoparticles	Au NPs (spheres, rods, and shells), Au NPs modified with TNF-*α*, TGF-*β*, or thio-PEG. Au NPs conjugated to T cells	DNA damage by ROS, mitochondrial dysfunction. Directs immune cells to tumor	No
Temozolomide (TMZ)	TMZ bound to nanocarriers (e.g., cucurbit[n]uril)	DNA alkylating agent	Yes
Carmustine (BCNU)	BCNU bound nanocomplex	DNA alkylating agent	No
Cisplatin	Liposomal cisplatin	Inorganic Pt^2+^ complexes DNA alkylating and intercalating agent	No
Irinotecan	Liposomal Irinotecan (e.g., CPX 1)	Inhibits DNA topoisomerase I & induces single strand DNA lesions	Yes
CRLX101	Polymeric NPs conjugated with camptothecin or bevacizumab	Antiangiogenic inhibitor of HIF-1	No
DaunoXome	Lipid encapsulated daunorubicin	Antimitotic by DNA intercalation and inhibits topoisomerase II	No
